# Resistance and clonal selection among Allium sativum L. germplasm resources to Delia antiqua M. and its correlation with allicin content

**DOI:** 10.1002/ps.5478

**Published:** 2019-06-09

**Authors:** Haiping Wang, Yahong Wu, Xiuhui Liu, Zhenzhen Du, Yang Qiu, Jiangping Song, Xiaohui Zhang, Xixiang Li

**Affiliations:** ^1^ Department of Genetic Resources, Institute of Vegetables and Flowers Chinese Academy of Agricultural Sciences Beijing China

**Keywords:** garlic, onion maggot, Delia antiqua M., Allium sativum L., resistance, allicin

## Abstract

**BACKGROUND:**

Garlic is the second largest allium crop after onion and is grown all over the world. The onion maggot (Delia antiqua M.) is a pest that seriously affects the yield and quality of garlic. Cultural controls and insecticides have several potential problems, including pesticide residue and development of resistance. Screening resistant varieties is an ideal alternative method.

**RESULTS:**

The resistance of 213 accessions of garlic clones against onion maggot was identified. The results showed that the pest index was between 5.56% and 91.11%, with classification into six groups by cluster analysis: HR (highly resistant), R (resistant), MR (moderately resistant), MS (moderately susceptible), S (susceptible) and HS (highly susceptible). Among these accessions, 9 and 30 were HR and R to onion maggot, respectively. Comparing the resistances of seven pairs of accessions between the original accessions and their progenies showed that single bulb clonal selection could be an effective way to improve allicin content, onion maggot resistance and most morphological traits. The relationship between allicin content and resistance was investigated, and a significant positive relationship was found. Accessions with a high content of allicin have great potential as resistant accessions.

**CONCLUSION:**

This study showed significant differences among garlic germplasm in their response to Delia antiqua M. Some accessions were highly resistant and tolerant. Utilization of these accessions will help minimize environmental pollution, preserve agro‐ecosystems and biodiversity, and make management processes more economical. Furthermore, these accessions could be used in breeding programs to develop new maggot‐resistant onion cultivars. © 2019 The Authors. Pest Management Science published by John Wiley & Sons Ltd on behalf of Society of Chemical Industry.

## 1 INTRODUCTION

Garlic (*Allium sativum* L.) has been cultivated for more than 5000 years and is presumed to have originated in central Asia,^1^ subsequently spreading west, south and east.^2^ Garlic is an important commercial crop that is widely grown around the world, especially in Asia and North Africa; China is the largest global producer, providing 80% of total world production, and the leading exporter.^3^, ^4^, ^5^, ^6^, ^7^ Following China, other significant garlic producers include India (5% of world production) and Bangladesh (1%). As of 2016, China produced 21 million tons.^8^ Garlic has been vegetatively propagated for millennia by planting cloves. Vegetative propagation results in additional diseases and pest problems throughout garlic developmental stages, causing considerable losses in yield. In addition to yield reductions, diseases and insect pests are also harmful to bulbs during harvesting, post‐harvest processing and marketing, lowering the quality of garlic and resulting in significant economic losses.^9^, ^10^

Onion maggot, *Delia antiqua*. M, is one of the most serious subterranean pests of scallion (*Allium fistulosum* L), garlic (*Allium sativum* L) and onion (*Allium cepa* L) in many regions of the world.^11^, ^12^, ^13^, ^14^, ^15^ The initial occurrence of onion maggot was reported in the USA in some regions of Wisconsin in the early twentieth century, and continues to cause serious damage in Asia, particularly China and Japan, Europe and North America.^16^ Although, the onion is the optimal host for this species, garlic can also serve as an important secondary host.^13^ In China, the root maggot, commonly known as the onion fly, is a hazardous soil pest that feeds on garlic.^17^ The larvae tend to aggregate in fields and directly damage plants. They mainly eat garlic roots, root necks, bulbs and seedlings, potentially leading to the death of the whole plant. Occasionally, the larvae can drill into the bulb and cause the bulb to rot. These insect pests have reportedly spread to much of the garlic‐producing areas of Qinghai, Xinjiang, Inner Mongolia, Shaanxi, Shanxi, Gansu, Ningxia, Liaoning, Hebei, Beijing, Henan, Jiangsu, and Shandong in China.^16^, ^17^ Furthermore, with climate change, highly suitable habitats for *D. antiqua* occur throughout most of East Asia, some regions of North America, Western Europe, and Western Asian countries near the Caspian Sea and the Black Sea.^15^ Strategic planning by agricultural organizations is required to identify regions that will need to develop integrated pest management (IPM) programs to manage the onion maggot.

The organic approach of crop rotation and breeding of varieties is the usual recommendations. Because of the household responsibility system for arable land in China, it is difficult to achieve timely rotation in practice. Because the larvae of the garlic maggot primarily damage the underground portions of plants, they are difficult to prevent or control. One of the most prevalent management practices against garlic maggot is the application of insecticides, such as organophosphates, carbamates and neonicotinoids. However, consistent use of chemicals to control pests not only poses a serious threat to the environment and people, but also slowly builds up pesticide resistance in pests. Most new‐generation pesticides are systemic in their mode of action, which may lead to a certain level of toxicity in the plant system and thus result in health hazards.^10^ Therefore, it is very important to explore and rationally exploit the pest‐resistance genes of plants. Selection of resistant varieties could be very promising and a few resistant garlic varieties against onion maggot have been selected out in the previous studies.^17^ To more efficiently use the germplasm collection, it is necessary to evaluate and characterize the preserved traits.^18^, ^19^, ^20^ Abundant garlic germplasm resources broaden the genetic variability and provide considerable opportunities for genetic research and breeding.^21^ The use of resistant cultivars is an important management tool to deal with disease and pest.^22^, ^23^, ^24^, ^25^, ^26^ Garlic is a typical vegetatively propagated crop, and clonal selection is the major breeding method. The large‐scale diversity of different ecotypes has been established over time in various areas of cultivation. According to incomplete statistics, > 2000 varieties are maintained in different countries. There are > 600 accessions of garlic germplasm in the national germplasm repository for vegetatively propagated vegetables in China. These abundant garlic germplasm resources broaden the genetic variability and provide considerable opportunities for genetic research and breeding.^27^ Pest‐resistant varieties could be selected from these extensive germplasm resources.

Different ecotypes of garlic display great morphological diversity and some traits are positively or negatively correlated with each other. In previous study, the only exception was for the garlic‐scape and bulb, the variety that grows higher had higher yield of garlic‐scape, and the variety that had wider diameter of pseudostem had higher yield of bulb.^28^ So, finding the relationship among traits and using fewer traits will make it easier to select target accessions with our character of interest for use in breeding programs.

Phytochemicals play an important role in plant resistance to insects.^29^, ^30^, ^31^ Some studies suggest that capsaicin,^32^ gingerol and garlic powder,^31^ and other substances with pungent odors, have obvious inhibitory effects on onion maggot. Garlic is generally very resistant to pests and is often grown specifically to protect neighboring plants. A number of studies have shown that sulfur‐containing organic compounds, such as thiosulfinates, play an important role in this function.^33^, ^34^, ^35^ Allicin is the most important functional component in garlic and has insecticidal and antibacterial effects. A previous study showed a positive correlation between the thiosulfinate content of garlic and resistance to *Bradysia odoriphaga* in a small garlic germplasm collection.^36^ In our previous studies, with a preliminary analysis based on 52 accessions of garlic, a high allicin content has great potential as a resistant material for breeding. However, the extent of the variation in the resistance of garlic cultivars to *Delia antiqua* M. and their allicin content, their relationship, and the potential to develop resistant varieties by clonal selection in a large germplasm collection from a whole country have scarcely been studied.

In this study, during the harvest period, variations in the pest resistance and allicin content of 213 accessions of garlic germplasm, covering the main garlic‐producing areas were evaluated in controlled field plots or in the laboratory. Correlations between resistance to the root maggot and allicin content in bulbs, and some morphological traits were investigated. The effect of clonal selection for resistance was investigated between seven pairs of accessions of clones. This study provides important germplasm resources and information for breeding resistant varieties of garlic.

## 2 MATERIALS AND METHODS

### 2.1 Garlic germplasm

A total of 213 accessions of garlic clones were used in this study. The samples, which were mainly collected from garlic‐producing areas in China, were provided by the national germplasm repository for vegetatively propagated vegetables (Table S1).

### 2.2 Progenies of single‐plant selection

accessions 8N017S, 8N036S, 8N141S, 8N167S, 8N254S, 8N257S and 8N261S were the progenies of the original accessions, i.e. 8N017, 8N036, 8N141, 8N167, 8N254, 8N257 and 8N261, obtained by single‐bulb selection and were propagated for 10 years beginning in 2006. The plants of the selected bulbs were healthy and resistant to *Delia antiqua* M.

### 2.3 Field experiments

Field experiments were conducted during 2014–2016 using a complete randomized block design with three replicates at same experiment field for several years. Each accession was planted by hand in a 4.5 m^2^ plot in four rows with 20 cm spacing between lines and 10 cm spacing within lines at the Vegetable Research Center of the International Agricultural High and New Technology Industrial Park, Chinese Academy of Agricultural Sciences. The experiment investigating morphological traits and allicin content was carried out in a normal field using normal field management. The experiment investigating pest index was carried out in the disease nursery and the plants were sufficiently watered, and no insecticides were applied.

### 2.4 Morphological trait data collection

Morphological trait data were collected according to previously described methods.^4^ Briefly, at various growth stages, the characteristics were measured according to the descriptors for garlic developed by the International Plant Genetic Resources Institute^36^ and descriptors and data standards for garlic by the Institute of Vegetables and Flowers, CAAS.^37^ In total, 21 quantitative and eight qualitative traits were investigated, and ten plants from each replicate were measured for quantitative traits from 2014 to 2016.

### 2.5 Allicin determination

Allicin determination was performed according to the method described in a previous study.^39^ Briefly, five bulbs and one clove from each bulb of each of the 213 accessions were randomly selected in three replicates, 40 days after harvest in 2016. The cloves were peeled to remove the dry protective leaves and stored at −20 °C for 3–4 h before chopping into small slices. The garlic slices were stored at −80 °C for 3–7 h, freeze‐dried, and finally ground to powder. Allicin content analysis was performed at the Supervision and Testing Center for Vegetable Quality, Ministry of Agriculture of China. The accuracy and stability of the methods were confirmed in a previous study and no replicates are necessary for allicin content detection.^39^ To confirm the accuracy, 40 randomly selected accessions of 213 accessions of garlic were detected in three replicates (Table S2).

### 2.6 Garlic resistance investigation

**Figure 1:**
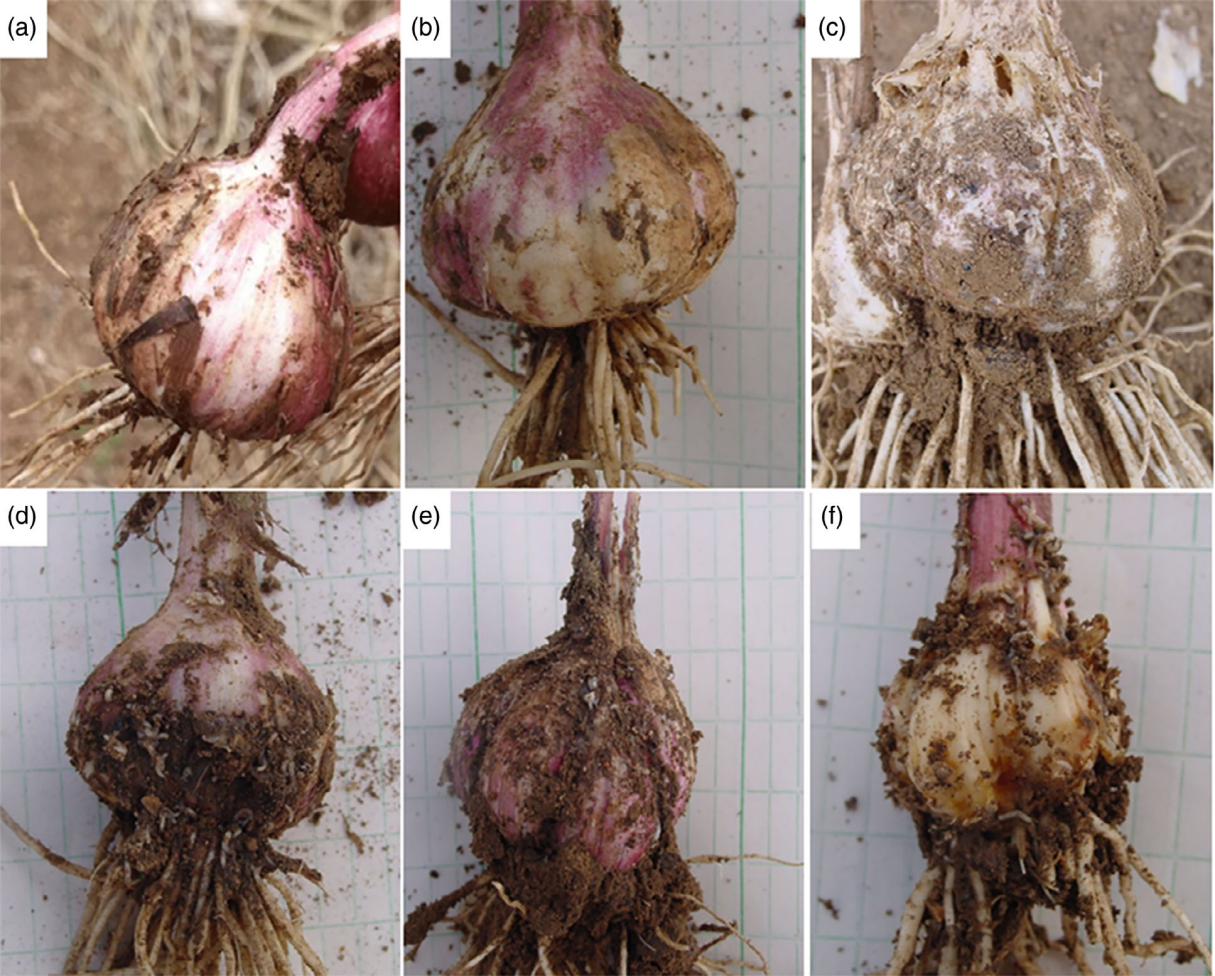
Reference photos for grades of resistance of individual plants to onion maggot (Delia antiqua M.): (a) grade 0; (b) grade 1; (c) grade 3; (d) grade 5; (e) grade 7; (f) grade 9.

The pest resistance of each accession was investigated according to the method described in a previous study.^17^ Eight individual plants of each replicate were randomly evaluated during the garlic harvest period in 2016. The grade for an individual plant is estimated by the numbers of larvae and the area of damage that the larvae fed upon. The grade for an individual plant was described as follows (Table 1): 0, no larvae, bulbs complete; 1, larvae present, damaged area of bulb was less than 1/4; 3, larvae present, damaged area of bulb was between 1/4 and 1/2, or there were > 30 larvae on the bulb; 5, damage was serious, damaged area of bulb was between 1/2 and 3/4, there were > 50 larvae on the bulb, and slight symptoms of rot were present; 7, damage was very serious, damaged area of bulb was greater than 3/4, although a few healthy cloves could be found, there were > 70 larvae on the bulb, and symptoms of rot were serious; 9, damage was very serious, all bulbs were damaged, there were no healthy cloves, and there were a large number of larvae. The investigator referred to pictures to code the grade for individual plants (Fig. 1). The pest index was calculated as: *PI* (%) = ∑ (*S i n i*)/9 N × 100, where *S i* is the grade, *n i* is the number of plants within a certain grade and *N* is the total number of plants investigated.

**Table 1:** Resistance distribution of the 213 accessions investigated in 2016

Resistance group	Pest index range	Accessions	Percent
HR	0–15	9	4.23
R	16–29	30	14.08
MR	30–39	58	27.23
MS	40–49	44	20.66
S	50–69	52	24.41
HS	70–100	20	9.39

HR, highly resistant to Delia antiqua M.; R, resistant (R) to Delia antiqua M.; MR, moderately resistant to Delia antiqua M.; S, susceptible to Delia antiqua M.; HS, highly susceptible to Delia antiqua M.

### 2.7 Data analysis

Data were analyzed using the software IBM SPSS statistics 25 (2017; SPSS Inc., Chicago, IL, USA). First, we tested the normality of the data for the pest index and allicin content using the Shapiro–Wilk test prior to statistical analysis. Data were transformed to normality using Blom's formula. Correlation analysis was performed between the pest index and allicin content with Pearson correlation coefficients. Nonparametric correlations among the pest index, allicin content and morphological traits were evaluated by Spearman correlation coefficients. To understand the distribution of the pest index among accessions, cluster analysis was performed based on the unweighted pair‐group method with arithmetic means (UPGMA), and the cluster tree diagram was constructed using the software Minitab 16 (2010; Minitab Inc., State College, PA, USA). To understand the effect of clonal selection activity on resistance, the resistances, allicin content and quantity traits of the progenies and their parent germplasms were compared.

## 3 RESULTS

### 3.1 Garlic resistance investigation and allicin content determination

**Figure 2:**
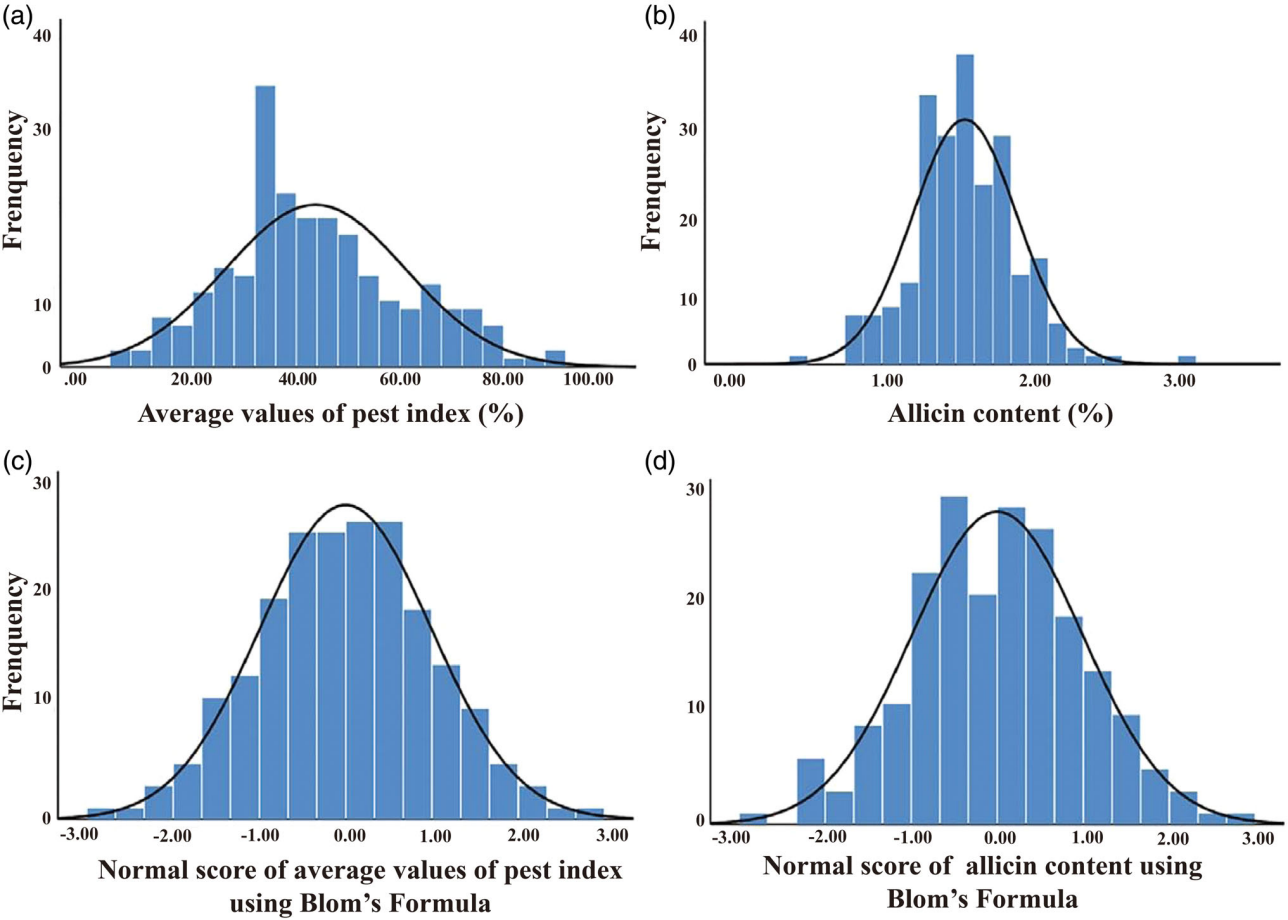
Distribution and normality of pest index and allicin content among 213 accessions of garlic investigated in 2016. Distribution of (a) average values of pest index; (b) rank average values of pest index after normality test using Blom's formula; (c) allicin content; and (d) rank allicin content after normality test using Blom's formula.

The pest resistance of each accession was investigated. The performance of different resistance grate was obvious (Fig. 1). There was wide variation in the resistance of the garlic germplasm to onion maggot (*Delia antiqua* M.), with pest index values ranging from 5.56% to 91.11% (Table S1). The frequency distribution chart of the pest index illustrated that the most pest index values were ranged from 20% to 80% and > 30% of accessions were ranged into scale from 26% to 28% (Fig. 2a).

The accuracy of the allicin detect methods was confirm by 40 accessions of garlic. Standard divisions between replicates were very small from 0.00 to 0.05 and one replicate for each accession should be acceptable (Table S2). Similar to the pest index, allicin content showed large variation among the germplasm from 0.45% to 3.01% (Table S1). The frequency distribution chart illustrated that the allicin content of most accessions of garlic was from 0.80% to 2.50% with only a few accessions > 3.00% or < 0.50% (Fig. 2b). Normality using the Shapiro–Wilk test indicated that data from both the pest index and allicin content did not meet the requirements of a standard normal distribution. The data were transformed to normality using Blom's formula (Fig. 2a and b). prior to statistical analysis.

### 3.2 Cluster analysis

**Figure 3:**
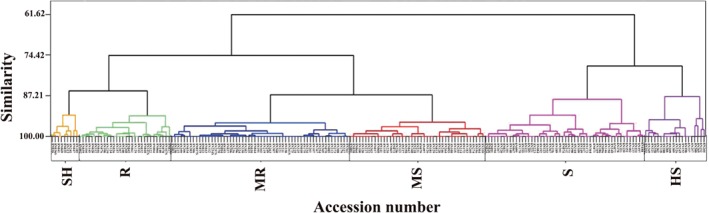
Tree dendrogram of the pest index values of 213 accessions of garlic. HR, highly resistant to Delia antiqua M.; R, resistant to Delia antiqua M.; MR, moderately resistant to Delia antiqua M.; S, susceptible to Delia antiqua M., HS, highly susceptible to Delia antiqua M.

According to the tree diagram and pest index, the 213 accessions of garlic were divided into six groups (Fig. 3 and Table 1). The first group, which was highly resistant (HR) to *Delia antiqua* M., included nine accessions, corresponding to 4.23% of the total accessions, with a pest index ranging from 0 to 15: 8N185 (Da Tai Suan), 8N047 (Qing Miao), 8N313 (Fu Kang Zi Suan), 8N035(Si, Liu Ban Suan), 8N241 (Hong Pi Suan), 8N041 (Zhong Mou Da Suan), 8N069 (Zao Hong Xuan), 8N36S Lai Wu Bai Pi) and 8N016 (Bang Zi Suan). The second group, which was resistant (R) to *Delia antiqua* M., included 30 accessions, corresponding to 14.08% of the total accessions, with a pest index ranging from 16 to 29. The third group, which was moderately resistant (MR) to *Delia antiqua* M., included 58 accessions, corresponding to 27.23% of the total accessions, with a pest index ranging from 30 to 39. The fourth group, which was moderately susceptible (MS) to *Delia antiqua* M., included 44 accessions, corresponding to 20.66% of the total accessions, with a pest index ranging from 40 to 49. The fifth group, which was susceptible (S) to *Delia antiqua* M., included 52 accessions, corresponding to 24.41% of the total accessions, with a pest index ranging from 50 to 69. The sixth group, which was highly susceptible (HS) to *Delia antiqua* M., included 20 accessions, corresponding to 9.39% of the total accessions, with a pest index ranging from 70 to 100.

### 3.3 The effect of selection on resistance

**Figure 4:**
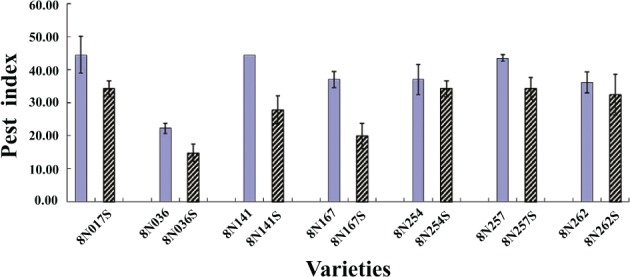
Effect of clonal selection on resistance improvement to Delia antiqua M investigated in 2016. 8N017, 8N036, 8N141, 8N167, 8N254, 8N257 and 8N261 are the original garlic germplasms; 8N017S, 8N036S, 8N141S, 8N167S, 8N254S, 8N257S and 8N261S are the corresponding offspring of the original garlic obtained by breeding activities. The bar values represent the mean ± SE.

Following single‐plant selection from natural populations, most progeny clones from the tested germplasms had significantly higher resistance than their parents (Fig. 4 and Table 2). The pest index of the progeny clone (8N017S) obtained by single‐plant selection from parent 8N017 was 34.26%, whereas that of its parent (8N017) was 44.44%. The pest index of the progeny clone (8N036S) obtained by single‐plant selection from parent 8N036 was 14.81%, whereas that of its parent (8N036) was 22.22%. In addition, significant differences in the pest index were observed between the progeny clone 8N141S (27.78%) obtained by single‐plant selection and its parent 8N141 (44.44%). Similarly, the pest indexes of the progeny clone 8N167S (19.19%) obtained by single‐plant selection and its parent 8N167 (37.04%) differed. Likewise, the pest index of the clone 8N167 (34.26%) obtained by single‐plant selection was found to be lower than that of its parent 8N167 (37.04%), and the pest index of the clone 8N257S (34.26%) obtained by single‐plant selection was found to be lower than that of its parent 8N257 (43.52%). Finally, the pest index of the progeny clone 8N261S (32.41%) derived by selection from its parent was lower than that of its parent 8N261 (36.11%). Cluster analysis also showed that parents and progeny could be grouped into different clusters (e.g. clone progeny 8N017S in Group MR and parent 8N017 in Group HS). Similarly, the clone progeny 8N036S appeared in Group HR, whereas its parent (8N036) was clustered into Group MR. Furthermore, the accession 8N141S appeared in Group R, whereas its parent (8N141) in Group S was susceptible to *Delia antiqua* M. The accession 8N167S appeared in Group R, whereas its parent (8N167) in Group MR was moderately resistant to *Delia antiqua* M. Although genotype 8N254S and its parent 8N254 both appeared in Group MR, the progeny 8N254S exhibited greater resistance than its parent, and similar results were obtained for 8N261S and its parent 8N261. The clone progeny 8N257S was clustered into Group MR and its parent 8N257 in Group S.

**Table 2:** Effect of clonal selection on resistance

Accession number	PI, mean ± SD	Cluster group
8N017	44.44 ± 9.62	HS
8N017S	34.26 ± 4.24	MR
8N036	22.22 ± 2.78	MR
8N036S	14.81 ± 4.46	HR
8N141	44.44 ± 0.00	S
8N141S	27.78 ± 7.35	R
8N167	37.04 ± 4.24	MR
8N167S	19.91 ± 6.56	R
8N254	37.04 ± 8.02	MR
8N254S	34.26 ± 4.24	MR
8N257	43.52 ± 1.60	S
8N257S	34.26 ± 5.78	MR
8N261	36.11 ± 5.56	MR
8N261S	32.41 ± 10.52	MR

PI, pest index; HR, highly resistant to Delia antiqua M.; R, resistant (R) to Delia antiqua M.; MR, moderately resistant to Delia antiqua M.; S, susceptible to Delia antiqua M.; HS, highly susceptible to Delia antiqua M.

**Figure 5:**
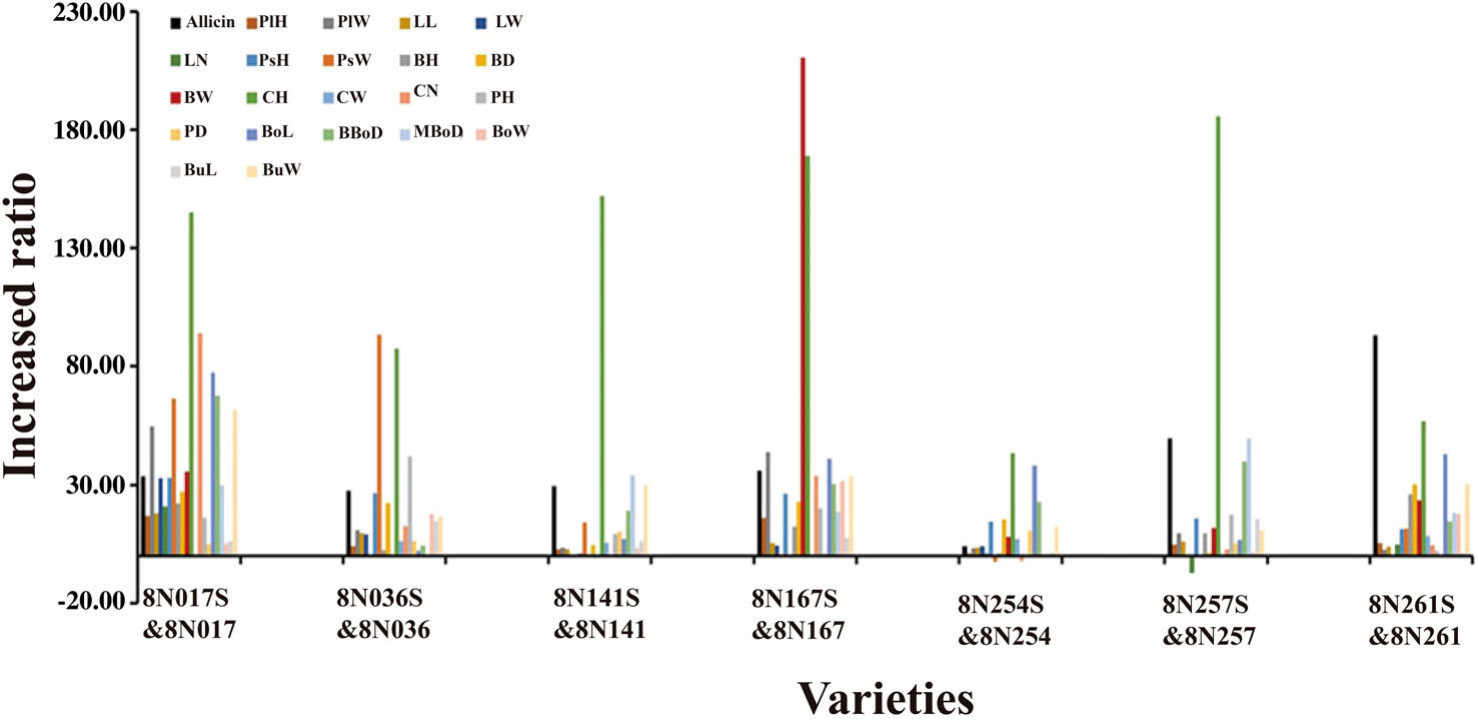
Effect of clonal selection on allicin content and 21 quantity traits compared between unselected and selected versions. Allcin, allicin; PlH, plant height; PlW, plant width; LL, leaf length; LW, leaf width; LN, leaf number; PsH, pseudostem height; PsW, pseudostem diameter; BH, bulb height; BD, bulb diameter; BW, bulb weight; CH, clove height; CW, clove diameter; CN, clove number; PH, thickness of shortened stem; PD, diameter of shortened stem; BoL, bolt length; BBoD, basal diameter of bolt; MBoD, mid‐diameter of bolt; BoW, weight per bolt; BuL, spathe length; BuW, spathe width.

The allicin content and 21 quantity traits were compared between unselected and selected versions of seven pairs of garlic clones., The results indicated that clonal selection was also very effective (Fig. 5). The allicin content and 20 morphological traits, except leaf number, of the selected versions were positively increased with a relative ratio from 0 to 200 plus compared with unselected versions. The above results demonstrated that clonal selection activity could be an effective way to improve pest resistance, allicin content and most of morphological traits in garlic.

### 3.4 Correlation coefficients among major plant morphological characteristics and resistance

Correlations between the pest index and allicin content were evaluated using both Pearson and Spearman correlation coefficients. A significant negative correlation was found between the pest index and allicin content according to the Pearson correlation coefficient (*r* = −0.787, Table 3), and identical results were obtained based on the Spearman correlation coefficient (Table 4). These results suggest that varieties with higher allicin content have greater resistance to *Delia antiqua* M.

**Table 3:** Spearman correlation coefficients between the pest index and allicin content for 213 accessions

		Pest index	Allicin content
Pest index	Pearson correlation	1	−0.787^a^
	Significance (two‐tailed)	–	0.000
	*N*	213	213
Allicin content	Pearson correlation	−0.787^a^	1
	Significance (two‐tailed)	0.000	–
	*N*	213	213

a Correlation is significant at the 0.01 level.

**Table 4:** Spearman correlation coefficients among morphological traits, allicin content and pest index for 213 accessions

Items	Pest index	Allicin content	Plant height	Plant width	Leaf length	Leaf width	Leaf number	Pseudostem height	Pseudostem diameter	Bulb height	Bulb diameter	Bulb weight	Clove height	Clove diameter	Clove number
Allicin content	−0.787**	–	–	–	–	–	–	–	–	–	–	–	–	–	–
Plant height	−0.098	0.087	–	–	–	–	–	–	–	–	–	–	–	–	–
Plant width	0.007	−0.043	0.453**	–	–	–	–	–	–	–	–	–	–	–	–
Leaf length	−0.004	−0.078	0.616**	0.734**	–	–	–	–	–	–	–	–	–	–	–
Leaf width	−0.063	−0.010	0.535**	0.534**	0.745**	–	–	–	–	–	–	–	–	–	–
Leaf number	−0.061	−0.038	0.410**	0.492**	0.525**	0.644**	–	–	–	–	–	–	–		–
Pseudostem height	−0.155*	0.119	0.697**	0.379**	0.501**	0.459**	0.464**	–	–	–	–	–	–	–	–
Pseudostem diameter	−0.053	−0.050	0.520**	0.424**	0.583**	0.731**	0.619**	0.438**	–	–	–	–	–	–	–
Bulb height	−0.191**	0.109	0.350**	0.172*	0.324**	0.485**	0.436**	0.417**	0.467**	–	–	–	–	–	–
Bulb diameter	−0.176*	0.100	0.416**	0.335**	0.457**	0.636**	0.507**	0.517**	0.526**	0.747**	–	–	–	–	–
Bulb weight	−0.133	0.088	0.456**	0.258**	0.432**	0.640**	0.544**	0.518**	0.592**	0.667**	0.817**	–	–	–	–
Clove height	−0.144*	0.031	0.015	−0.056	0.035	0.174*	0.177*	0.034	0.155*	0.189**	0.155*	0.298**	–	–	–
Clove diameter	−0.115	0.103	0.197**	0.060	0.002	−0.059	0.071	0.197**	0.097	0.252**	0.222**	0.121	0.021	–	–
Clove number	−0.065	−0.004	0.240**	0.253**	0.369**	0.551**	0.356**	0.336**	0.365**	0.386**	0.584**	0.591**	0.079	−0.399**	–
Basal plate thickness	−0.102	0.041	0.265**	0.266**	0.297**	0.292**	0.271**	0.379**	0.282**	0.289**	0.353**	0.171*	−0.045	0.069	0.223**
Basal plate diameter	−0.175*	0.123	0.303**	0.425**	0.420**	0.420**	0.385**	0.462**	0.292**	0.362**	0.558**	0.411**	−0.055	0.053	0.457**
Scape length	−0.048	0.067	0.231**	0.250**	0.094	0.043	0.126	0.323**	−0.016	0.024	0.194**	0.125	−0.069	0.121	0.059
Basal diameter of scape	0.099	−0.131	0.326**	0.277**	0.332**	0.364**	0.222**	0.182**	0.288**	0.141*	0.243**	0.275**	0.094	0.020	0.183**
Mid‐diameter of scape	0.053	−0.089	0.304**	0.293**	0.323**	0.412**	0.291**	0.193**	0.330**	0.175*	0.287**	0.325**	0.114	0.020	0.219**
Scape weight	0.003	−0.001	0.326**	0.246**	0.273**	0.376**	0.219**	0.228**	0.276**	0.167*	0.294**	0.325**	0.054	0.038	0.171*
Spathe length	0.038	−0.094	0.325**	0.440**	0.482**	0.329**	0.288**	0.211**	0.236**	−0.027	0.073	0.078	−0.033	−0.072	0.153*
Spathe width	0.114	−0.074	0.070	0.058	0.068	0.185**	−0.002	−0.027	0.108	−0.037	0.104	0.054	−0.021	0.012	0.075
Plant type	0.091	−0.147*	−0.065	0.060	0.152*	0.204**	0.109	0.034	0.109	0.082	0.191**	0.162*	0.043	−0.002	0.172*
Leaf posture	−0.051	0.107	0.146*	0.016	−0.018	−0.103	−0.062	0.058	−0.034	−0.032	−0.164*	−0.127	−0.091	0.105	−0.210**
Leaf color	−0.039	0.003	0.153*	0.058	0.135*	0.130	0.083	0.193**	0.126	0.162*	0.188**	0.237**	−0.031	0.041	0.169*
Leaf wax	−0.039	0.030	0.101	−0.050	−0.006	0.085	0.111	0.099	0.209**	0.110	0.133	0.266**	0.172*	0.138*	0.023
Bolting	0.054	−0.032	0.189**	0.369**	0.293**	0.273**	0.119	0.207**	0.084	−0.058	0.163*	0.083	−0.103	−0.115	0.234**
Bulb type	0.009	0.014	−0.193**	−0.077	−0.082	−0.222**	−0.216**	−0.254**	−0.147*	−0.181**	−0.278**	−0.275**	−0.102	0.123	−0.270**
Bulb color	−0.013	0.000	−0.029	0.090	0.039	−0.020	0.054	0.002	0.009	−0.051	0.015	−0.026	−0.192**	0.129	−0.074
Clove type	−0.021	0.008	0.083	0.045	−0.058	−0.015	0.100	0.146*	0.000	0.029	0.081	−0.008	0.073	0.021	0.154*

Items	Basal plate thickness	Basal plate diameter	Scape length	Basal diameter of scape	Mid‐diameter of scape	Scape weight	Spathe length	Spathe width	Plant type	Leaf posture	Leaf color	Leaf wax	Bolting	Bulb type	Bulb color
Basal plate diameter	0.399**	–	–	–	–	–	–	–	–	–	–	–	–	–	–
Scape length	0.154*	0.308**	–	–	–	–	–	–	–	–	–	–	–	–	–
Basal diameter of scape	0.047	0.200**	0.580**	–	–	–	–	–	–	–	–	–	–	–	–
Mid‐diameter of scape	0.107	0.248**	0.552**	0.922**	–	–	–	–	–	–	–	–	–	–	–
Scape weight	0.127	0.272**	0.621**	0.817**	0.876**	–	–	–	–	–	–	–	–	–	–
Spathe length	0.203**	0.266**	0.394**	0.484**	0.459**	0.465**	–	–	–	–	–	–	–	–	–
Spathe width	0.050	0.114	0.380**	0.641**	0.690**	0.750**	0.314**	–	–	–	–	–	–	–	–
Plant type	0.000	0.091	0.016	0.178**	0.188**	0.135*	−0.033	0.260**	–	–	–	–	–	–	–
Leaf posture	−0.044	−0.044	−0.055	−0.138*	−0.162*	−0.072	0.089	−0.142*	−0.682**	–	–	–	–	–	–
Leaf color	−0.001	0.096	−0.026	0.069	0.067	0.029	−0.056	−0.069	0.041	−0.044	–	–	–	–	–
Leaf wax	−0.160*	−0.095	−0.012	0.047	0.024	0.001	−0.166*	−0.035	0.073	−0.097	0.140*	–	–	–	–
Bolting	0.247**	0.353**	0.515**	0.520**	0.526**	0.508**	0.506**	0.524**	0.169*	−0.106	−0.090	−0.164*	–	–	–
Bulb type	−0.090	−0.151*	−0.243**	−0.253**	−0.277**	−0.239**	−0.072	−0.163*	−0.094	0.147*	−0.193**	0.025	−0.172*	–	–
Bulb color	0.099	−0.006	0.074	0.075	0.088	0.087	0.124	0.053	−0.008	0.039	−0.072	−0.117	0.085	0.067	–
Clove type	0.024	0.140*	0.079	−0.035	−0.023	−0.036	−0.008	−0.033	0.025	0.020	0.107	0.029	0.008	−0.135*	−0.313**

** Correlation is significant at the 0.01 level.

* Correlation is significant at the 0.05 level.

In addition, in the correlation matrix (Table 3), some traits showed high correlations with each other, indicating that some traits could be selected for a variety of improvement programs to reduce time and labor. Plant width was positively correlated with leaf length with a correlation coefficient of 0.734, and leaf length was positively correlated with leaf width with a correlation coefficient of 0.745. Bulb diameter was positively correlated with bulb weight with a correlation coefficient of 0.667. Basal diameter of scape was positively correlated with mid‐diameter of scape with a correlation coefficient of 0.922. Basal diameter of scape was positively correlated with scape weight with a correlation coefficient of 0.817.

## 4 DISCUSSION

This investigation revealed differences in resistance levels among a large‐scale garlic germplasm and identified resistant resources. Cluster analysis based on morphological traits of genetic resources facilitate breeding based on an understanding of the germplasm and the selection of potential material among a large‐scale accession with high efficiency, as shown in studies of the genetic resources of many crops,^40^, ^41^, ^42^ including horticultural crops such as pepper,^43^, ^44^ cowpea,^45^ watermelon,^46^ brassica,^47^ radish,^15^ and garlic.^7^ In this study, we used cluster analysis to classify 213 accessions of garlic collections into six groups, including HR, R, MR, MS, S, and HS, according to the pest index, facilitating the identification of resistance among the germplasm. Nine accessions were highly resistant (HR) to *Delia antiqua* M.: 8N185 (Da Tai Suan), 8N047 (Qing Miao), 8N313 (Fu Kang Zi Suan), 8N035 (Si, Liu Ban Suan), 8N241 (Hong Pi Suan), 8N041 (Zhong Mou Da Suan), 8N069 (Zao Hong Xuan), 8N36S (Lai Wu Bai Pi) and 8N016 (Bang Zi Suan). The results were consistent with our previous study in that highly resistant 8N313 (Fu Kang Zi Suan 8N041 (Zhong Mou Da Suan), 8N069 (Zao Hong Xuan) were confirmed in this study.^17^ Zhu^39^ found that Zhongmu garlic was sensitive to *B. odoriphaga*; however, we found that 8N041 (Zhong Mou Da Suan) was highly resistant to *Delia antiqua* (Meigen). These differences may be due to regional variations or differences in planting conditions.^36^ The utilization of these cultivars will help minimize environmental pollution, preserve agro‐ecosystems and biodiversity, and make management processes more economical. Furthermore, these cultivars could be used in breeding programs to develop new cultivars that are resistant to onion maggot.

The allicin in garlic bulbs might be an effective factor against *D. antiqua* M. In our previous study,^17^ we evaluated differences in resistance to *D. antiqua* M. among 34 garlic cultivars in the field and found that a cultivar with high allicin content exhibited strong resistance. Other previous studies also speculated that sulfides, the decomposition products of thiosulfinate, possess greater insecticidal activity.^36^ The allicin content in garlic bulbs was confirmed positively by the resistance of garlic in the disease nursery. However, whether allicin plays a dominant role in resistance requires further study with bioactivity identification. Besides, some traits showed high correlations with each other, indicating that some traits could be selected for variety improvement programs to reduce time and labor.

The discovery and selection of mutants in natural populations is an important breeding activity for improving the allicin content in bulbs and the resistance of plants to *D. antiqua* M. Allicin content is affected by both geographical and genetic factors.^48^, ^49^, ^50^ Although the allicin contents determined in different studies are not comparable, several authors^51^, ^52^, ^53^ have agreed that extensive genetic variation in allicin content exists. In the previous study, the allicin content of 213 accessions of garlic grown in the same environment ranged from 0.45% to 3.01%, which could be mainly ascribed to the influence of genetics. Clonal selection could obviously be used to improve allicin content in garlic bulbs.^4^ In the present study, upon selection from natural populations, most progeny clones from the tested germplasms had significantly higher resistance than their parent materials, showing an obvious effect of single‐plant selection. Breeding programs could improve the resistance of garlic. We speculate that natural mutations might accumulate throughout the generations of garlic, and resistant mutant genes could be screened by breeding activities.

This study provides a foundation for breeding pest‐resistant varieties of garlic. However, the results were obtained in disease nursery experiments, and the mechanism of garlic resistance to *Delia antiqua* M. requires further study with bioactivity and molecular identification.

## 5 CONCLUSIONS

The findings of the study showed significant differences among garlic germplasm in their response to *Delia antiqua* M. Some accessions were found to be highly resistant. Utilization of these accessions will help to minimize environmental pollution, preserve the agro‐ecosystems and biodiversity, and make management processes more economical. Furthermore, these accessions could be used in breeding programs to develop new resistant cultivars to onion maggot. Clonal selection activity could be an effective way to improve pest resistance, allicin content and most of morphological traits in garlic.

## Supporting information


**Table S1.** Origins, pest index and allicin content of 213 accessions of experimental materials
**Table S2.** Accuracy of allicin detect method confirmed by 40 accessions

## References

[1] Vavilov NI , The origin, variation, immunity and breeding of cultivated plants. Soil Sci 72:482 (1951).

[2] Etoh T , Watanabe H and Iwai S , RAPD variation of garlic clones in the center of origin and the westernmost area of distribution. Mem Fac Agric Kogoshima Univ 37:21–27 (2001).

[3] Wang H , Li X , Shen D , Yang O and Song J , Diversity evaluation of morphological traits and allicin content in garlic (Allium sativum L.) from China. Euphytica 198:243–254 (2014).

[4] Wang H , Li X , Liu X , Oiu Y , Song J and Zhang X , Genetic diversity of garlic (Allium sativum L.) germplasm from China by fluorescent‐based AFLP, SSR and InDel markers. Plant Breeding 135:743–750 (2016).

[5] Srivastava SC , Sharma UC , Singh BK and Yadava HS , A profile of garlic production in India: facts, trends and opportunities. Int J Agric Environ Biotechnol 5:477–482 (2012).

[6] Etoh T and Simon PW , Diversity, fertility and seed production of garlic, in Allium Crop Science: Recent Advances, Vol. 5, ed. by Rabinowitch HD and Currah L, CABI *publishing*, New York, NY 100016, USA pp. 101–117 (2002).

[7] Panthee DR , KC RB , Regmi HN , Subedi PP , Bhattarai S and Dhakal J , Diversity analysis of garlic (Allium sativum L.) germplasms available in Nepal based on morphological characters. Genet Resour Crop Evol 53:205–212 (2006).

[8] Wikipedia , *Garlic Production in China* Available: https://en.wikipedia.org/wiki/Garlic_production_in_China [11 March 2019].

[9] Mcdonald MR , Jaime MDLA and Hovius MHY , Management of diseases of onions and garlic. Dis Fruits Vegetables 2:149–200 (2004).

[10] MR K , JR K , Kumar D , SP R and Singh A , Management of major diseases and insect pests of onion and garlic: a comprehensive review. J Plant Breeding Crop Sci 6:160–170 (2014).

[11] Ellis PR and Eckenrode CJ , Factors influencing resistance in *Allium* sp. to onion maggot. Bull Entomol Soc Am 25:151–153 (1979).

[12] Zhang YX , Wang Z , Mao‐Wen SU , Zhao HP , Xiao‐Dan MA and Xue M , Selective studies on *Delia antique* (Meigen) to 4 host plants. China Vegetables 4:83–86 (2012).

[13] Zhang H , Wu S , Wang S and Lei Z , Effect of *Beauveria bassiana* on the activity of defense enzymes and cellular defense response of adult of Delia antiqua (Meigen). Chin J Biol Control 33:198–205 (2017).

[14] Ning S , Zhang W , Sun Y and Feng J , Development of insect life tables: comparison of two demographic methods of Delia antiqua (Diptera: Anthomyiidae) on different hosts. Sci Rep 7:4821 (2017). 28684791 10.1038/s41598-017-05041-5 PMC5500477

[15] Lu H , Yu Q , Han H , Owen MJ and Powles SB , A novel *psbA* mutation (Phe274–Val) confers resistance to PSII herbicides in wild radish (Raphanus raphanistrum). Pest Manag Sci 75:144–151 (2019). 29797480 10.1002/ps.5079

[16] Ning S , Wei J and Feng J , Predicting the current potential and future world wide distribution of the onion maggot, Delia antiqua using maximum entropy ecological niche modeling. PLoS One 12:e0171190 (2017). 28158259 10.1371/journal.pone.0171190 PMC5291381

[17] Wang H , Li X , Shen D , Yang Q and Song J , Identification of resistance of Allium sativum L. germplasm resources to *Delia antique* M. J Plant Genet Resour 11:578–582 (2010).

[18] Anumalla M , Roychowdhury R , Geda CK , Mazid M and Rathoure AK , Utilization of plant genetic resources and diversity analysis tools for sustainable crop improvement with special emphasis on Rice. Int J Adv Res 3:1155–1175 (2015).

[19] Upadhyaya HD and Gowda CLL , Enhancing utilization of plant genetic resources in crop improvement, in Plant breeding in post genomics era Proceedings of Second National Plant Breeding Congress, 1–3 March, 2006, India*,* (2006).

[20] Eunice OE , Nwosu DJ , Alamu O , Coker DO and Aladele SE , Trends in genetic resources utilization in Nigeria national gene bank. Int J Conserv Sci 6:209–216 (2015).

[21] Wang XQ , Kwon SW and Park YJ , Evaluation of genetic diversity and linkage disequilibrium in Korean‐bred rice varieties using SSR markers. Electron J Biotechnol 16:1–20 (2013).

[22] Vieira SS , Bueno AF , Boff MI , Bueno RC and Hoffman‐Campo CB , Resistance of soybean genotypes to Bemisia tabaci (Genn.) *Biotype B* (Hemiptera: Aleyrodidae). Neotrop Entomol 40:117–122 (2011). 21437493 10.1590/s1519-566x2011000100018

[23] Sah DN and Bonman JM , Effects of seedbed management on blast development in susceptible and partially resistant rice cultivars. J Phytopathol 136:73–81 (2010).

[24] Asano K and Tamiya S , Breeding of pest and disease resistant potato cultivars in Japan by using classical and molecular approaches. Japan Agric Res Q 50:1–6 (2016).

[25] Avnery S , Mauzerall DL and Fiore AM , Increasing global agricultural production by reducing ozone damages via methane emission controls and ozone‐resistant cultivar selection. Glob Chang Biol 19:1285–1299 (2013). 23504903 10.1111/gcb.12118 PMC3627305

[26] Didelot F , Sustainable management of scab control through the integration of apple resistant cultivars in a low‐fungicide input system. Agr Ecosyst Environ 217:41–48 (2016).

[27] Zhao WG , Chung JW , Lee GA , Ma KH , Kim HH , Kim KT *et al*, Molecular genetic diversity and population structure of a selected core set in garlic and its relatives using novel SSR markers. Plant Breeding 130:46–54 (2011).

[28] Kong SP , Sun JQ , Xiong WU , Yang YY , Huo YM and Kun XU , Analysis of relationship between variations of Main agronomic traits and yield in garlic. Sci Agric Sin 48:1240–1248 (2015).

[29] Bagchi VA , Siegel JP , Demkovich MR , Zehr LN and Berenbaum MR , Impact of pesticide resistance on toxicity and tolerance of hostplant phytochemicals in *Amyelois transitella* (Lepidoptera: Pyralidae). J Insect Sci 16:1–7 (2016). 27620560 10.1093/jisesa/iew063 PMC5019020

[30] Mukandiwa L , Naidoo V and Katerere DR , The use of Clausena anisata in insect pest control in Africa: a review. J Ethnopharmacol 194:1103–1111 (2016). 27836776 10.1016/j.jep.2016.11.002

[31] Thibout E , Lecomte C and Auger J , Substances soufrées des *Allium* et insectes. Acta Bot Gallica 143:137–142 (2013).

[32] Gurung T , Techawongstien S , Suriharn B and Techawongstien S , Impact of environments on the accumulation of Capsaicinoids in *Capsicum* spp. HortScience 46:1576–1581 (2011).

[33] Awan UA , Ali S , Shahnawaz AM , Shafique I , Zafar A , Khan MAR *et al*, Biological activities of *Allium sativum* and Zingiber officinale extracts on clinically important bacterial pathogens, their phytochemical and FT‐IR spectroscopic analysis. Pak J Pharm Sci 30:729–745 (2017). 28653916

[34] Nchu F , Magano SR and Eloff JN , Repellent activities of dichloromethane extract of Allium sativum (garlic) (Liliaceae) against *Hyalomma rufipes* (Acari). J S Afr Vet Assoc 87:e1–e5 (2016). 10.4102/jsava.v87i1.1356 PMC6138159 28155295

[35] Kim S , Kim D‐B , Jin W , Park J , Yoon W , Lee Y *et al*, Comparative studies of bioactive organosulphur compounds and antioxidant activities in garlic (Allium sativum L.), elephant garlic (Allium ampeloprasum L.) and onion (Allium cepa L.). Nat Prod Res 32:1193–1197 (2018). 28475377 10.1080/14786419.2017.1323211

[36] Zhu G , Luo Y , Xue M , Zhou F , Zhao H , Ji G *et al*, Resistance of garlic cultivars to Bradysia odoriphaga and its correlation with garlic thiosulfinates. Sci Rep 7:1–11 (2017). 28607407 10.1038/s41598-017-03617-9 PMC5468350

[37] IPGRI , Descriptors for Allium (Allium spp.). International Plant Genetic Resources Institute, Nairobi, Kenya (2001).

[38] Li X , Discriptors and Data Standard for Garlic (Allium sativum L.). China Agriculture Press, Beijing, China (2006).

[39] Wang HP , Li XX , Liu XY , SHEN D , Qiu Y , Song JP *et al*, Allicin UPLC detection technology and Tts utilization in garlic genetic resources. J Plant Genet Resour 13:936–945 (2012).

[40] Singh P and Jaiswal JP , Assessment of genetic diversity in bread wheat (Triticum aestivum L. em. Thell), genotypes based on agro‐morphological traits using Mahalanobis D2 statistic. Environ Ecol 31:679–682 (2013).

[41] Saeed F , Farooq J , Mahmood A , Riaz M , Hussain T and Majeed A , Assessment of genetic diversity for cotton leaf curl virus (CLCuD), fiber quality and some morphological traits using different statistical procedures in Gossypium hirsutum L. Aust J Crop Sci 8:442–447 (2017).

[42] Golabadi M , Golkar P and Shahsavari MR , Genetic analysis of agro‐morphological traits in promising hybrids of sunflower (Helianthus annuus L.). Acta Agric Slov 105:249–260 (2015).

[43] Sisaphaithong T , Genetic diversity and clustering of tomato [Lycopersicon lycopersicum (L.) Karsten] accessions in Laos based on morphological traits (vol 34, pg 12, 2009). Philipp J Crop Sci 34:112–112 (2009).

[44] Zewdie Y , Tong NK and Bosland P , Establishing a core collection of capsicum using a cluster analysis with enlightened selection of accessions. Genet Res Crop Evol 51:147–151 (2004).

[45] Simango K and Lungu D , Evaluation of physiological and morphological traits conferring drought tolerance in cowpea, in Proceedings of the Second RUFORUM Biennial Regional Conference on Building Capacity for Food Security in Africa, 20–24 September 2010, ed. by Adipala E, Tusiime G and Majaliwa Mwanjololo JG RUFORM, Kampala, Uganda Entebbe, Uganda, pp. 441–445 (2010).

[46] Yang X , Ren R , Rumiana R , Xu J , Li P , Man Z *et al*, Genetic diversity and population structure of core watermelon (Citrullus lanatus) genotypes using DArTseq‐based SNPs. Plant Genet Resour 14:226–233 (2016).

[47] Wright AJ , Back MA , Stevens M and Sparkes DL , Evaluating resistant brassica trap crops to manage Heterodera schachtii (Schmidt) infestations in eastern England. Pest Manag Sci 75:438–443 (2019). 29998541 10.1002/ps.5134

[48] Baghalian K , Naghavi MR , Ziai SA and Badi HN , Post‐planting evaluation of morphological characters and allicin content in Iranian garlic (Allium sativum L.) ecotypes. Sci Hortic 107:405–410 (2006).

[49] Baghalian K , Sanei MR , Naghavi MR , Khalighi A and Badi HAN , Post‐culture evaluation of morphological divergence in Iranian garlic ecotypes, in Proceedings of the IVth International Symposium on Edible Alliaceae, ed by Guangshu L, ISHS, International Society for Horticultural Science, Leuven pp. 123–128 (2005).

[50] Mostafa HHA , Wang HP , Liu XY and Li XX , Impact of genetic factor and geographical location on Allicin content of garlic (Allium sativum) germplasm from Egypt and China. Int J Agric Biol 17:156–162 (2015).

[51] Vargas S , Ioset KN , Hay AE , Ioset JR , Wittlin S and Hostettmann K , Screening medicinal plants for the detection of novel antimalarial products applying the inhibition of beta‐hematin formation. J Pharm Biomed Anal 56:880–886 (2011). 21872416 10.1016/j.jpba.2011.06.026

[52] Mirzaei R , Liaghati H and Damghani AM , Evaluating yield quality and quantity of garlic as affected by different farming systems and garlic clones. Pak J Biol Sci 10:2219–2224 (2007). 19070185 10.3923/pjbs.2007.2219.2224

[53] Hirata S , Abdelrahman M , Yamauchi N and Shigyo M , Characteristics of chemical components in genetic resources of garlic Allium sativum collected from all over the world. Genet Resour Crop Evol 63:1–11 (2016).

