# Supplementary material for: Resistance and clonal selection among Allium sativum L. germplasm resources to Delia antiqua M. and its correlation with allicin content
Source: Pest Manag Sci. 2019 Jun 9;75(10):2830–9. doi: 10.1002/ps.5478 (PMC6771725; doi:10.1002/ps.5478)
Supplement: Supplementary file 1 — Table S1. Origins, pest index and allicin content of 213 accessions of experimental materials Table S2. Accuracy of allicin detect method confirmed by 40 accessions [file PS-75-2830-s001.doc]

# Supplementary information:

# Resistance and clonal selection among *Allium sativum* L*.* Germplasm Resources to *Delia antiqua* M. and Its Correlation with Allicin Content

**Haiping Wang***,

Yahong Wu,

Xiuhui Liu,

Zhenzhen Du,

Yang Qiu,

Jiangping Song,

Xiaohui Zhang,

Xixiang Li

Institute of Vegetables and Flowers, Chinese Academy of Agricultural Sciences, 12 Nandajie, Zhongguancun, Haidian District, Beijing 100081, China.

Supplements

Table S1**.**  Origins, pest index and allicin content of 213 accessions of experimental materials

| Serial No. | Accession ID | Origin | PI±SE | Allicin content(%) |
| --- | --- | --- | --- | --- |
| 1 | 8N001 | Sichuan, China | 43.52 ±11.23 | 1.53 |
| 2 | 8N002 | Sichuan, China | 25.00 ±7.35 | 2.03 |
| 3 | 8N003 | Sichuan, China | 31.48 ±4.24 | 1.82 |
| 4 | 8N004 | Sichuan, China | 18.06 ±8.67 | 2.21 |
| 5 | 8N010 | Sichuan, China | 36.11 ±5.56 | 1.64 |
| 6 | 8N013 | Sichuan, China | 45.37 ±1.60 | 1.46 |
| 7 | 8N016 | Sichuan, China | 15.28 ±7.73 | 2.26 |
| 8 | 8N017 | Sichuan, China | 44.44 ±9.62 | 2.06 |
| 9 | 8N019 | Hubei, China | 34.26 ±4.24 | 1.85 |
| 10 | 8N023 | Hubei, China | 34.26 ±4.24 | 1.76 |
| 11 | 8N024 | Hubei, China | 50.00 ±12.73 | 2.06 |
| 12 | 8N025 | Hubei, China | 33.80 ±15.36 | 1.73 |
| 13 | 8N026 | Hubei, China | 50.00 ±12.11 | 1.47 |
| 14 | 8N027 | Jiangxi, China | 15.74 ±1.60 | 2.21 |
| 15 | 8N028 | Jiangxi, China | 24.07 ±4.24 | 2.00 |
| 16 | 8N030 | Heilongjiang, China | 33.33 ±4.81 | 1.73 |
| 17 | 8N031 | Shanghai, China | 50.93 ±4.24 | 1.47 |
| 18 | 8N032 | Shanxi, China | 28.70 ±3.21 | 2.06 |
| 19 | 8N034 | Beijing, China | 29.63 ±3.21 | 1.85 |
| 20 | 8N035 | Shandong, China | 11.11 ±0.00 | 2.21 |
| 21 | 8N036 | Shandong, China | 22.22 ±2.78 | 1.99 |
| 22 | 8N037 | Shandong, China | 35.19 ±3.21 | 1.65 |
| 23 | 8N038 | Shandong, China | 43.52 ±4.24 | 1.51 |
| 24 | 8N039 | Shandong, China | 25.00 ±2.78 | 1.94 |
| 25 | 8N040 | Shandong, China | 28.70 ±3.21 | 2.00 |
| 26 | 8N041 | Shandong, China | 13.89 ±0.00 | 2.38 |
| 27 | 8N042 | Shandong, China | 40.74 ±8.49 | 1.55 |
| 28 | 8N043 | Shandong, China | 34.26 ±3.21 | 1.81 |
| 29 | 8N044 | Shandong, China | 33.33 ±2.78 | 1.81 |
| 30 | 8N045 | Shandong, China | 21.30 ±4.24 | 2.06 |
| 31 | 8N046 | Shandong, China | 26.85 ±1.60 | 1.85 |
| 32 | 8N047 | Shandong, China | 7.41 ±0.00 | 2.21 |
| 33 | 8N060 | Shanxi, China | 44.44 ±7.35 | 1.65 |
| 34 | 8N061 | Shanxi, China | 23.15 ±11.23 | 2.00 |
| 35 | 8N064 | Shanxi, China | 43.52 ±8.49 | 1.49 |
| 36 | 8N065 | Shanxi, China | 41.67 ±4.81 | 1.37 |
| 37 | 8N066 | Shanxi, China | 18.52 ±1.60 | 2.07 |
| 38 | 8N067 | Shanxi, China | 52.38 ±0.00 | 1.37 |
| 39 | 8N069 | Shanxi, China | 13.89 ±3.67 | 2.60 |
| 40 | 8N070 | Shanxi, China | 39.15 ±0.46 | 1.58 |
| 41 | 8N071 | Shanxi, China | 65.28 ±5.89 | 1.26 |
| 42 | 8N072 | Shanxi, China | 62.96 ±4.24 | 1.44 |
| 43 | 8N073 | Shanxi, China | 41.67 ±2.78 | 1.63 |
| 44 | 8N074 | Shanxi, China | 61.11 ±13.89 | 1.34 |
| 45 | 8N076 | Xinjiang, China | 25.93 ±1.60 | 1.73 |
| 46 | 8N078 | Thailand | 18.98 ±7.65 | 2.07 |
| 47 | 8N079 | Thailand | 39.81 ±3.21 | 1.58 |
| 48 | 8N084 | Shanxi, China | 53.70 ±5.78 | 1.33 |
| 49 | 8N085 | Thailand | 38.89 ±4.81 | 1.67 |
| 50 | 8N091 | Guangdong, China | 50.00 ±5.56 | 1.44 |
| 51 | 8N093 | Shanxi, China | 47.22 ±0.00 | 1.24 |
| 52 | 8N094 | Liaoning, China | 34.72 ±1.96 | 1.68 |
| 53 | 8N096 | Gansu, China | 63.89 ±2.78 | 1.18 |
| 54 | 8N097 | Sichuan, China | 39.81 ±15.30 | 1.63 |
| 55 | 8N099 | Xicang, China | 52.78 ±2.78 | 1.34 |
| 56 | 8N100 | Shanghai, China | 43.52 ±8.93 | 1.83 |
| 57 | 8N101 | Sichuan, China | 50.93 ±1.60 | 1.29 |
| 58 | 8N102 | Heilongjiang, China | 76.85 ±6.42 | 2.21 |
| 59 | 8N104 | Shanxi, China | 25.00 ±4.81 | 1.47 |
| 60 | 8N107 | Sichuan, China | 36.57 ±6.56 | 1.63 |
| 61 | 8N108 | Guangdong, China | 47.22 ±3.93 | 1.34 |
| 62 | 8N109 | Sichuan, China | 57.41 ±9.76 | 1.31 |
| 63 | 8N112 | Hubei, China | 34.26 ±4.24 | 1.31 |
| 64 | 8N113 | Hubei, China | 42.59 ±4.24 | 1.54 |
| 65 | 8N115 | Shanxi, China | 47.22 ±2.78 | 1.27 |
| 66 | 8N116 | Sichuan, China | 55.56 ±7.35 | 1.31 |
| 67 | 8N117 | Guizhou, China | 58.33 ±5.56 | 1.27 |
| 68 | 8N118 | Shanxi, China | 74.07 ±4.24 | 1.54 |
| 69 | 8N120 | Shanxi, China | 43.52 ±4.24 | 1.53 |
| 70 | 8N121 | Jiangsu, China | 32.41 ±1.60 | 1.83 |
| 71 | 8N122 | Jiangsu, China | 51.85 ±6.42 | 1.42 |
| 72 | 8N123 | Jiangsu, China | 54.63 ±6.99 | 1.33 |
| 73 | 8N124 | Jiangsu, China | 25.46 ±0.80 | 1.99 |
| 74 | 8N126 | Jiangsu, China | 39.81 ±3.21 | 1.58 |
| 75 | 8N127 | Jiangsu, China | 42.59 ±9.76 | 1.54 |
| 76 | 8N128 | Jiangsu, China | 45.37 ±5.78 | 1.53 |
| 77 | 8N129 | Jiangsu, China | 23.15 ±1.60 | 1.99 |
| 78 | 8N130 | Anhui, China | 31.48 ±4.24 | 1.83 |
| 79 | 8N139 | Anhui, China | 67.59 ±6.99 | 1.13 |
| 80 | 8N140 | Jiangxi, China | 18.52 ±5.61 | 1.51 |
| 81 | 8N141 | Shandong, China | 44.44 ±0.00 | 1.42 |
| 82 | 8N145 | Sichuan, China | 24.54 ±6.26 | 2.06 |
| 83 | 8N146 | Hebei, China | 64.44 ±0.00 | 1.26 |
| 84 | 8N148 | Neimenggu, China | 50.93 ±4.24 | 1.26 |
| 85 | 8N149 | Neimenggu, China | 57.41 ±10.52 | 1.29 |
| 86 | 8N150 | Neimenggu, China | 64.81 ±6.99 | 1.22 |
| 87 | 8N151 | Neimenggu, China | 67.59 ±5.78 | 1.08 |
| 88 | 8N152 | Neimenggu, China | 71.30 ±4.24 | 1.09 |
| 89 | 8N154 | Shandong, China | 69.44 ±2.78 | 1.13 |
| 90 | 8N155 | Shandong, China | 44.44 ±2.78 | 1.51 |
| 91 | 8N156 | Shandong, China | 49.07 ±10.52 | 1.48 |
| 92 | 8N157 | Jiangsu, China | 22.22 ±5.56 | 2.06 |
| 93 | 8N167 | Yunnan, China | 37.04 ±4.24 | 1.44 |
| 94 | 8N168 | Yunnan, China | 40.74 ±1.60 | 1.66 |
| 95 | 8N169 | Yunnan, China | 65.74 ±4.24 | 1.09 |
| 96 | 8N170 | Yunnan, China | 52.78 ±12.11 | 1.48 |
| 97 | 8N172 | Yunnan, China | 46.30 ±4.24 | 1.63 |
| 98 | 8N173 | Heilongjiang, China | 58.33 ±0.00 | 1.22 |
| 99 | 8N175 | Yunnan, China | 36.11 ±5.56 | 1.71 |
| 100 | 8N178 | Hubei, China | 62.96 ±12.83 | 1.44 |
| 101 | 8N179 | Hebei, China | 37.96 ±11.23 | 1.33 |
| 102 | 8N180 | Hubei, China | 74.07 ±3.21 | 1.11 |
| 103 | 8N181 | Hubei, China | 39.81 ±6.42 | 1.62 |
| 104 | 8N182 | Hubei, China | 67.59 ±5.78 | 1.09 |
| 105 | 8N183 | Hubei, China | 46.30 ±15.30 | 1.48 |
| 106 | 8N185 | Guizhou, China | 5.56 ±1.39 | 3.01 |
| 107 | 8N186 | Guizhou, China | 65.74 ±21.03 | 1.94 |
| 108 | 8N188 | Guizhou, China | 68.98 ±10.61 | 1.17 |
| 109 | 8N189 | Gansu, China | 55.56 ±11.11 | 1.32 |
| 110 | 8N190 | Gansu, China | 71.30 ±4.24 | 1.17 |
| 111 | 8N191 | Gansu, China | 33.33 ±2.78 | 1.27 |
| 112 | 8N192 | Sichuan, China | 53.70 ±5.78 | 1.33 |
| 113 | 8N193 | Sichuan, China | 34.72 ±1.96 | 1.95 |
| 114 | 8N194 | Jiangxi, China | 50.00 ±4.81 | 1.26 |
| 115 | 8N197 | Gansu, China | 69.44 ±2.78 | 0.99 |
| 116 | 8N198 | Gansu, China | 25.93 ±6.42 | 1.95 |
| 117 | 8N200 | Henan, China | 82.67 ±4.35 | 0.89 |
| 118 | 8N201 | Gansu, China | 56.48 ±4.24 | 1.31 |
| 119 | 8N202 | Ningxia, China | 33.33 ±4.81 | 1.82 |
| 120 | 8N205 | Gansu, China | 51.85 ±15.30 | 1.42 |
| 121 | 8N206 | Hebei, China | 35.19 ±6.99 | 1.67 |
| 122 | 8N207 | Hebei, China | 22.22 ±0.00 | 1.53 |
| 123 | 8N208 | Hebei, China | 74.07 ±4.24 | 1.47 |
| 124 | 8N209 | Liaoning, China | 74.07 ±4.24 | 0.96 |
| 125 | 8N211 | Ningxia, China | 32.41 ±3.21 | 1.78 |
| 126 | 8N212 | Jiangsu, China | 38.89 ±8.33 | 1.61 |
| 127 | 8N215 | India | 31.48 ±4.24 | 1.84 |
| 128 | 8N217 | Jiangsu, China | 43.52 ±6.99 | 1.51 |
| 129 | 8N218 | Jiangsu, China | 40.74 ±5.78 | 1.57 |
| 130 | 8N219 | Shandong, China | 33.33 ±0.00 | 1.77 |
| 131 | 8N220 | Shandong, China | 44.44 ±2.78 | 1.53 |
| 132 | 8N221 | Shandong, China | 51.85 ±0.00 | 1.42 |
| 133 | 8N222 | Shandong, China | 43.52 ±1.60 | 1.52 |
| 134 | 8N223 | Shandong, China | 51.85 ±1.60 | 1.47 |
| 135 | 8N231 | Hubei, China | 40.74 ±5.78 | 1.56 |
| 136 | 8N232 | Hubei, China | 88.89 ±2.78 | 0.83 |
| 137 | 8N233 | Hubei, China | 67.59 ±6.99 | 1.22 |
| 138 | 8N234 | Hubei, China | 36.11 ±2.78 | 1.64 |
| 139 | 8N236 | Hubei, China | 35.19 ±3.21 | 1.68 |
| 140 | 8N237 | Jiangsu, China | 71.30 ±10.52 | 1.04 |
| 141 | 8N238 | Jiangsu, China | 32.41 ±1.60 | 1.73 |
| 142 | 8N239 | Jiangsu, China | 25.93 ±1.60 | 1.60 |
| 143 | 8N240 | Jiangsu, China | 64.81 ±6.99 | 1.26 |
| 144 | 8N241 | Yunnan, China | 12.50 ±3.67 | 2.28 |
| 145 | 8N244 | Yunnan, China | 91.11 ±0.00 | 0.45 |
| 146 | 8N245 | Yunnan, China | 75.00 ±5.56 | 0.93 |
| 147 | 8N246 | Yunnan, China | 45.37 ±11.23 | 1.53 |
| 148 | 8N248 | Yunnan, China | 22.22 ±11.11 | 1.99 |
| 149 | 8N249 | Yunnan, China | 33.33 ±0.82 | 1.78 |
| 150 | 8N254 | Yunnan, China | 37.04 ±8.02 | 1.42 |
| 151 | 8N255 | Yunnan, China | 47.22 ±0.00 | 1.48 |
| 152 | 8N256 | Xinjiang, China | 41.67 ±3.93 | 1.54 |
| 153 | 8N257 | Jiangsu, China | 71.30 ±5.78 | 1.02 |
| 154 | 8N258 | Jiangsu, China | 42.59 ±3.21 | 1.61 |
| 155 | 8N259 | Jiangsu, China | 31.02 ±11.31 | 1.84 |
| 156 | 8N260 | Jiangsu, China | 46.30 ±8.49 | 1.47 |
| 157 | 8N261 | Jiangsu, China | 36.11 ±5.56 | 0.83 |
| 158 | 8N263 | Jiangsu, China | 33.33 ±4.81 | 1.78 |
| 159 | 8N264 | Shandong, China | 45.37 ±11.56 | 1.51 |
| 160 | 8N265 | Jiangsu, China | 86.11 ±2.78 | 1.61 |
| 161 | 8N266 | Jiangsu, China | 30.56 ±4.81 | 1.84 |
| 162 | 8N267 | Jiangsu, China | 50.00 ±12.11 | 1.47 |
| 163 | 8N268 | Jiangsu, China | 22.22 ±1.61 | 1.99 |
| 164 | 8N269 | Jiangsu, China | 79.63 ±4.24 | 0.83 |
| 165 | 8N270 | Jiangsu, China | 34.26 ±6.99 | 1.26 |
| 166 | 8N271 | Jiangsu, China | 61.11 ±12.73 | 1.26 |
| 167 | 8N272 | Jiangsu, China | 60.19 ±4.24 | 1.26 |
| 168 | 8N273 | Jiangsu, China | 38.89 ±0.00 | 1.78 |
| 169 | 8N274 | Jiangsu, China | 46.30 ±5.78 | 1.48 |
| 170 | 8N275 | Jiangsu, China | 32.41 ±8.02 | 1.61 |
| 171 | 8N296 | Yunnan, China | 35.19 ±3.21 | 1.57 |
| 172 | 8N298 | Yunnan, China | 59.72 ±5.89 | 1.26 |
| 173 | 8N302 | Yunnan, China | 35.65 ±5.61 | 1.64 |
| 174 | 8N303 | Yunnan, China | 29.63 ±9.76 | 1.84 |
| 175 | 8N304 | Yunnan, China | 49.07 ±6.42 | 1.47 |
| 176 | 8N306 | Yunnan, China | 29.63 ±4.24 | 0.83 |
| 177 | 8N307 | Yunnan, China | 33.33 ±2.78 | 1.78 |
| 178 | 8N308 | Yunnan, China | 38.89 ±2.78 | 1.61 |
| 179 | 8N309 | Yunnan, China | 74.07 ±5.78 | 0.89 |
| 180 | 8N310 | Yunnan, China | 32.41 ±1.60 | 1.82 |
| 181 | 8N312 | Yunnan, China | 24.07 ±4.24 | 1.99 |
| 182 | 8N313 | Xinjiang, China | 10.42 ±0.98 | 1.84 |
| 183 | 8N314 | Xinjiang, China | 47.22 ±4.81 | 1.47 |
| 184 | 8N315 | Yunnan, China | 23.15 ±6.99 | 2.03 |
| 185 | 8N318 | Yunnan, China | 59.26 ±8.93 | 1.26 |
| 186 | 8N320 | Yunnan, China | 50.00 ±5.56 | 1.48 |
| 187 | 8N321 | Yunnan, China | 78.70 ±11.23 | 0.83 |
| 188 | 8N322 | Yunnan, China | 28.24 ±6.85 | 1.75 |
| 189 | 8N324 | Yunnan, China | 35.19 ±1.60 | 1.64 |
| 190 | 8N325 | Yunnan, China | 32.41 ±3.21 | 1.78 |
| 191 | 8N326 | Yunnan, China | 73.15 ±6.42 | 0.89 |
| 192 | 8N327 | Yunnan, China | 37.96 ±3.21 | 1.61 |
| 193 | 8N329 | Yunnan, China | 39.81 ±3.21 | 1.57 |
| 194 | 8N330 | Yunnan, China | 35.19 ±5.78 | 1.57 |
| 195 | 8N351 | Spain | 62.04 ±8.93 | 1.23 |
| 196 | 8N352 | Spain | 54.63 ±1.60 | 1.32 |
| 197 | 8N355 | Yunnan, China | 52.78 ±9.62 | 1.34 |
| 198 | 8N358 | Korea | 78.70 ±3.21 | 1.84 |
| 199 | 8N359 | Korea | 48.15 ±8.93 | 1.47 |
| 200 | 8N360 | Korea | 33.33 ±14.43 | 1.82 |
| 201 | 8N361 | Korea | 37.90 ±2.53 | 1.64 |
| 202 | 8N362 | Korea | 77.78 ±2.78 | 0.83 |
| 203 | 8N363 | Europe | 43.52 ±1.60 | 1.48 |
| 204 | 8N364 | Europe | 58.33 ±2.78 | 1.26 |
| 205 | 8N365 | Europe | 37.04 ±3.21 | 1.64 |
| 206 | 8N367 | Europe | 54.63 ±12.83 | 1.61 |
| 207 | 8N141S | Shandong, China | 27.78 ±7.35 | 1.84 |
| 208 | 8N167S | Yunnan, China | 19.91 ±6.56 | 1.96 |
| 209 | 8N017S | Sichuan, China | 34.26 ±4.24 | 1.54 |
| 210 | 8N254S | Yunnan, China | 34.26 ±4.24 | 1.28 |
| 211 | 8N257S | Jiangsu, China | 34.26 ±5.78 | 1.53 |
| 212 | 8N261S | Jiangsu, China | 32.41 ±10.52 | 1.60 |
| 213 | 8N036S | Shandong, China | 14.81 ±4.46 | 1.56 |

Table S2**.**  Accuracy of allicin detect method confirmed by 40 accessions

|  | Replicates | | |  |  |
| --- | --- | --- | --- | --- | --- |
| Accession ID | I | II | III | Average | SE |
| 8N003 | 1.84 | 1.83 | 1.77 | 1.82 | 0.04 |
| 8N013 | 1.49 | 1.42 | 1.46 | 1.46 | 0.04 |
| 8N017 | 2.04 | 2.04 | 2.10 | 2.06 | 0.03 |
| 8N019 | 1.80 | 1.90 | 1.85 | 1.85 | 0.05 |
| 8N025 | 1.69 | 1.77 | 1.73 | 1.73 | 0.04 |
| 8N028 | 2.03 | 1.95 | 2.03 | 2.00 | 0.05 |
| 8N030 | 1.74 | 1.72 | 1.73 | 1.73 | 0.01 |
| 8N042 | 1.53 | 1.57 | 1.54 | 1.55 | 0.02 |
| 8N043 | 1.82 | 1.82 | 1.79 | 1.81 | 0.01 |
| 8N060 | 1.66 | 1.63 | 1.65 | 1.65 | 0.02 |
| 8N065 | 1.38 | 1.36 | 1.37 | 1.37 | 0.01 |
| 8N072 | 1.43 | 1.44 | 1.46 | 1.44 | 0.02 |
| 8N073 | 1.64 | 1.62 | 1.63 | 1.63 | 0.01 |
| 8N074 | 1.31 | 1.39 | 1.30 | 1.34 | 0.05 |
| 8N093 | 1.24 | 1.20 | 1.26 | 1.24 | 0.03 |
| 8N107 | 1.60 | 1.65 | 1.64 | 1.63 | 0.03 |
| 8N113 | 1.56 | 1.49 | 1.57 | 1.54 | 0.04 |
| 8N121 | 1.83 | 1.79 | 1.89 | 1.83 | 0.05 |
| 8N127 | 1.56 | 1.51 | 1.56 | 1.54 | 0.03 |
| 8N130 | 1.88 | 1.82 | 1.81 | 1.83 | 0.04 |
| 8N139 | 1.13 | 1.11 | 1.13 | 1.13 | 0.01 |
| 8N141 | 1.40 | 1.43 | 1.42 | 1.42 | 0.01 |
| 8N169 | 1.11 | 1.08 | 1.08 | 1.09 | 0.01 |
| 8N170 | 1.50 | 1.47 | 1.47 | 1.48 | 0.01 |
| 8N172 | 1.58 | 1.68 | 1.64 | 1.63 | 0.05 |
| 8N175 | 1.71 | 1.71 | 1.71 | 1.71 | 0.00 |
| 8N189 | 1.28 | 1.32 | 1.35 | 1.32 | 0.03 |
| 8N190 | 1.13 | 1.22 | 1.14 | 1.17 | 0.05 |
| 8N192 | 1.38 | 1.32 | 1.29 | 1.33 | 0.04 |
| 8N205 | 1.40 | 1.44 | 1.41 | 1.42 | 0.02 |
| 8N207 | 1.50 | 1.57 | 1.54 | 1.53 | 0.03 |
| 8N212 | 1.64 | 1.61 | 1.58 | 1.61 | 0.03 |
| 8N221 | 1.42 | 1.44 | 1.40 | 1.42 | 0.02 |
| 8N222 | 1.53 | 1.55 | 1.47 | 1.52 | 0.04 |
| 8N231 | 1.56 | 1.56 | 1.55 | 1.56 | 0.00 |
| 8N254 | 1.42 | 1.44 | 1.40 | 1.42 | 0.02 |
| 8N256 | 1.55 | 1.54 | 1.54 | 1.54 | 0.00 |
| 8N265 | 1.61 | 1.59 | 1.64 | 1.61 | 0.02 |
| 8N322 | 1.71 | 1.74 | 1.79 | 1.75 | 0.04 |
| 8N351 | 1.26 | 1.21 | 1.21 | 1.23 | 0.03 |
